# Hsp70 chaperone blocks α-synuclein oligomer formation *via* a novel engagement mechanism

**DOI:** 10.1016/j.jbc.2021.100613

**Published:** 2021-03-30

**Authors:** Jiahui Tao, Amandine Berthet, Y. Rose Citron, Paraskevi L. Tsiolaki, Robert Stanley, Jason E. Gestwicki, David A. Agard, Lisa McConlogue

**Affiliations:** 1 Department of Biochemistry and Biophysics, University of California San Francisco, San Francisco, California, USA; 2 Gladstone Institute of Neurological Disease, The Gladstone Institutes, San Francisco, California, USA; 3 Department of Pharmaceutical Chemistry, Institute for Neurodegenerative Diseases and UCSF Weill Institute for Neurosciences, University of California San Francisco, San Francisco, California, USA

**Keywords:** α-synuclein, chaperone, Hsp70, oligomer, Parkinson's disease, protein folding, Lewy body dementia, synucleinopathy, AMPNP, adenosine-5'-[(α,β)-imido]diphosphate, ASyn, α-synuclein, BiP, binding immunoglobulin protein, CHIP, C terminus of Hsc70-interacting protein, gLuc, Gaussia luciferase, Hsc70, constitutive 70-KDa cytosolic heat shock molecular chaperone, Hsp70, inducible 70-KDa cytosolic heat shock molecular chaperone, Hsp/c70s, 70-KDa cytosolic heat shock molecular chaperones, LBD, Lewy body dementias, LgBit, large portion of nLuc, NBD, nucleotide-binding domain, nLuc, NanoBit luciferase, PD, Parkinson’s disease, PPIs, protein–protein interactions, SBD, substrate-binding domain, SEC-MALS, combined size-exclusion chromatography and multiangle light scattering, SmBit, the small portion of nLuc, ThT, thioflavin T

## Abstract

Overexpression and aggregation of α-synuclein (ASyn) are linked to the onset and pathology of Parkinson’s disease and related synucleinopathies. Elevated levels of the stress-induced chaperone Hsp70 protect against ASyn misfolding and ASyn-driven neurodegeneration in cell and animal models, yet there is minimal mechanistic understanding of this important protective pathway. It is generally assumed that Hsp70 binds to ASyn using its canonical and promiscuous substrate-binding cleft to limit aggregation. Here we report that this activity is due to a novel and unexpected mode of Hsp70 action, involving neither ATP nor the typical substrate-binding cleft. We use novel ASyn oligomerization assays to show that Hsp70 directly blocks ASyn oligomerization, an early event in ASyn misfolding. Using truncations, mutations, and inhibitors, we confirm that Hsp70 interacts with ASyn *via* an as yet unidentified, noncanonical interaction site in the C-terminal domain. Finally, we report a biological role for a similar mode of action in H4 neuroglioma cells. Together, these findings suggest that new chemical approaches will be required to target the Hsp70-ASyn interaction in synucleinopathies. Such approaches are likely to be more specific than targeting Hsp70’s canonical action. Additionally, these results raise the question of whether other misfolded proteins might also engage Hsp70 *via* the same noncanonical mechanism.

Neuropathological, biochemical, and genetic evidence strongly implicates α-Synuclein (ASyn) in the onset and progression of Parkinson’s disease (PD) and related synucleinopathies including Lewy body dementias (LBD), multiple systems atrophy, and Alzheimer’s disease. Aggregated ASyn inclusions are a hallmark of these diseases (1), and ASyn gene mutations or multiplications cause early onset PD or LBD (2, 3, 4). The sequential misfolding of ASyn into oligomers and fibrils is central to the pathogenesis of the synucleinopathies. ASyn gene multiplications and mutations causing PD and LBD are associated with enhanced oligomer and membrane-associated fibril formation (5, 6, 7, 8, 9). Significant evidence indicates that misfolded ASyn seeding and spread throughout the brain underlie disease progression (10, 11). The severity of disease correlates with the progressive spread of aggregated ASyn in patients (12), and misfolding is associated with toxicity in cell and animal models (13). Although central to disease, ASyn misfolding has been challenging to target therapeutically.

A potential approach might be to target cellular chaperones that mitigate protein misfolding (14). The constitutive (Hsc70) and inducible (Hsp70) forms of the 70-KDa cytosolic heat shock molecular chaperones (Hsp/c70s) assist a wide variety of folding processes and provide broad protection against protein misfolding in the cell. Hsp70 polymorphisms are associated with PD (15) and in PD patients, stress-induced Hsp70 accumulates in a thwarted attempt to clear aggregated ASyn (16). Although Hsp70 is protective against ASyn pathogenicity in cell and animal models (17, 18, 19, 20, 21, 22), little is known about its protective mechanisms. The general biochemical mechanisms of Hsp70 action are well understood. Hsp/c70s are composed of a nucleotide-binding domain (NBD), which has ATPase activity, and a substrate-binding domain (SBD), which contains the canonical binding cleft for misfolded proteins. In addition, Hsp/c70s have disordered, C-terminal regions that engage in additional protein–protein interactions (PPIs). Hsp/c70s have two known mechanisms of mitigating protein misfolding: an ATP-cycling dependent “foldase” action, which is able to restore damaged proteins, and an ATP-independent “holdase” mode, which binds to unfolded proteins to prevent aggregation (23). Although in concert with cochaperones Hsp70 can disaggregate ASyn fibrils in an ATP-cycling mode (24, 25), Hsp/c70s block ASyn fibrillization (26, 27, 28, 29, 30, 31, 32, 33) in an ATP-independent manner (27, 28, 29, 30, 31, 32), indicating a “holdase”-based mechanism. It has been assumed that this holdase-like activity was mediated *via* Hsp/c70’s canonical substrate-binding site in the SBD, which is required for other known holdase roles (34). Unfortunately, the promiscuous engagement of the canonical substrate-binding site with a broad array of Hsp70 substrates, in either ATP-dependent or holdase modes, has precluded targeting Hsp70’s canonical actions as an effective therapeutic approach for neurodegenerative diseases.

As shown for other amyloid misfolding proteins, compelling evidence supports prefibrillar ASyn oligomers, and not fibrillar deposits, as the pathogenic species in disease (5, 35, 36, 37, 38, 39). ASyn oligomers are directly toxic to cells (39), and mutations enhancing ASyn oligomer formation increase ASyn toxicity in neurons and rodents (35, 36, 37). Hsp70 leads to reduced ASyn oligomer levels in cells (19, 40). However, a direct impact of Hsp70 on ASyn oligomerization has not been determined as this has only been indirectly surmised using ASyn fibrillization endpoint assays (26, 27, 28, 30, 31). We therefore investigated the direct impact of Hsp70 on ASyn oligomerization and its mechanism of action, using novel ASyn oligomerization biochemical assays. We show that Hsp70 directly blocks ASyn oligomerization in an ATP-independent holdase manner. However, unlike other known holdase mechanisms (34), we unexpectedly found that Hsp70’s activity was not mediated by the canonical Hsp70 substrate-binding site. Recently, a few examples of noncanonical Hsp70 interactions have been discovered (41), but we further found that these noncanonical sites are not involved. Finally, we found that Hsp70 blocking of ASyn oligomer formation is mediated by a similar mechanism in cells as well. Together, these results indicate the presence of a previously unidentified noncanonical interaction site. These findings have major implications for the use of Hsp70 as a drug target in synucleinopathies.

## Results

### A novel biochemical assay detecting ASyn oligomerization

**Figure 1:**
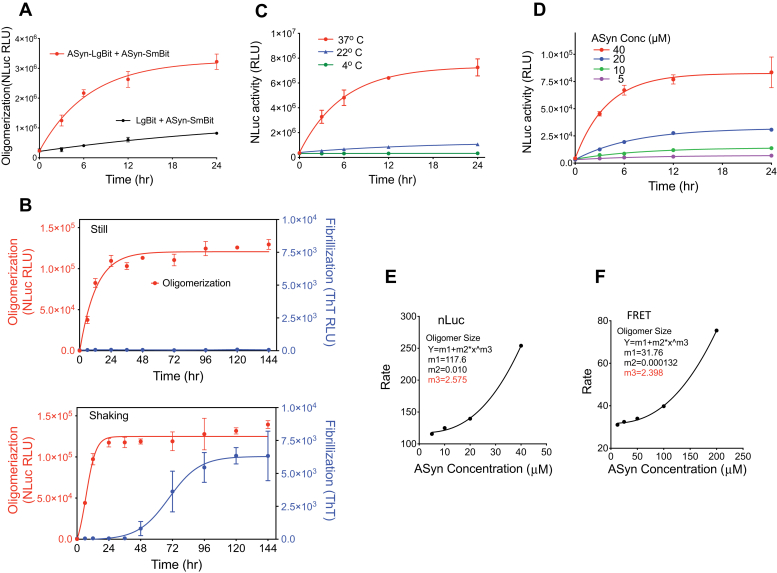
**ASyn biochemical oligomerization assays.***A*, split nLuc-tagged ASyn biochemical oligomerization assay development. LgBiT protein alone (*black*) or ASyn protein tagged with LgBit (ASyn-LgBiT, *red*) is mixed with ASyn protein tagged with SmBit (ASyn-SmBiT) at 10 μM each, incubated without shaking at 37 °C, and assayed for nLuc activity at various times. Oligomerization is detected by complementation of the split tags attached to ASyn. *B*, in total, 10 μM each of ASyn-LgBiT and ASyn-SmBiT is incubated in PBS under still (*top panel*) or shaking (*bottom panel*) conditions and analyzed for either ASyn oligomerization using the nLuc assay (*red*) or fibrillization *via* ThT fluorescence (*blue*). Oligomerization did not require shaking, whereas fibrillization did. *C*, temperature dependence of split nLuc-tagged ASyn oligomerization assay. Tagged ASyn was incubated at various temperatures and nLuc activity measured. 37 °C was required for robust oligomer formation. *D*, various concentrations of ASyn-LgBiT and ASyn-SmBiT were assayed for ASyn oligomerization using the nLuc assay. Oligomerization is dependent on ASyn concentration. *E*, initial rates of ASyn oligomerization in the nLuc assay, calculated from the data in *D*, are plotted against ASyn concentration and analyzed by the formula Y = m1 + m2∗xˆm3 to derive a minimal size of detected ASyn oligomer (m3) of 2.6 ASyn molecules. *F*, initial rates of ASyn oligomerization in the FRET assay, calculated from the data in Fig. S4, are plotted against ASyn concentration. Analysis as in E derived a minimal size of detected ASyn oligomer (m3) of 2.4 ASyn molecules. In all graphs the symbols show mean and bars show ±s.d., n = 3.

Because oligomers are the likely pathologic species, we focused on Hsp70’s action on ASyn oligomer formation. In prior studies, we established biochemical ASyn oligomerization assays that are based on either the complementation of split Gaussia luciferase (gLuc) or FRET between fluorophores on separate ASyn molecules (42). Both of these assays have limitations. The FRET assay has a restricted dynamic range and requires extended incubation times (Compare ASyn concentration response of nLuc Fig. 1*E* to FRET Fig. 1*F*, Fig. S4) (42). We find that in cells the gLuc tags drive ASyn into large aggregates raising questions about possible artifactual tag effects in this system (Fig. S1). We therefore developed an improved biochemical complementation assay using split NanoBit luciferase (nLuc) (43) tags placed on separate ASyn molecules (Fig. 1). Importantly, unlike either split fluorescent proteins (such as split GFP) or the Gaussia split luciferase (44), the nLuc complementation system employs fully reversible and quite weakly interacting components that are stable and nonlinkable *via* disulfide bonds (43). Purified ASyn species fused at its C-terminus with either the large (LgBit) or the small (SmBit) portion of nLuc were incubated together, and the formation of oligomers was monitored by reconstituted nLuc activity. Split nLuc tags placed on separate ASyn molecules reconstituted luciferase activity, whereas removal of ASyn from one of the tags gave minimal background signal (Fig. 1*A*). Oligomerization assays can be complicated by the heterogeneity of misfolded assemblies produced. It is important to avoid agitation, which leads to formation of higher-order fibrillar aggregates (45). This has been a particular problem with investigation of Hsp70 interactions with ASyn misfolding as ATP-dependent binding of Hsp/c70s to ASyn fibrils aggregates Hsp70 (29), competes effectively with chaperone engagement with soluble ASyn (32), and impairs Hsp/c70s blockage of fibrillization (30, 32). The formation of oligomers in the nLuc assay here did not require the agitation needed for the formation of thioflavin T (ThT) reactive fibrillar species (Fig. 1*B*). Furthermore, the oligomers detected did not have the characteristic lag phase seen in ASyn fibrillization reactions, which is consistent with early-stage oligomer formation.

The oligomerization reaction also showed a strict dependence on temperature, with efficient formation observed at 37 °C and negligible formation at room or lower temperatures (Fig. 1*C*). The highly quantitative split nLuc assay also allowed us to monitor the reaction’s dependence on ASyn concentration (Fig. 1*D*), providing insight into the number of ASyn monomers that must assemble to reconstitute luciferase activity (Fig. 1*E*). The data showed that the minimal assembly required to detect an nLuc signal was between dimers or trimers (n= 2.6; Fig. 1*E*), which matched well with what we saw in the absence of complementation tags using a FRET based assay (n = 2.4; Fig. 1*F*). nLuc signal developed at approximately fourfold lower ASyn concentrations than the FRET signal (Fig. 1*E* *versus* Fig. 1*F*), either a consequence of the inherent avidity intrinsic to any complementation system, even one with low tag affinities such as the nLuc system (43), or a consequence of having a tag (46). This increased sensitivity is expected to be beneficial when exploring the effects of oligomerization modulators. Importantly, split-tagged ASyn behaved like untagged ASyn in cells as the split nLuc tags did not drive ASyn into large aggregates (Fig. S1).

**Figure 2:**
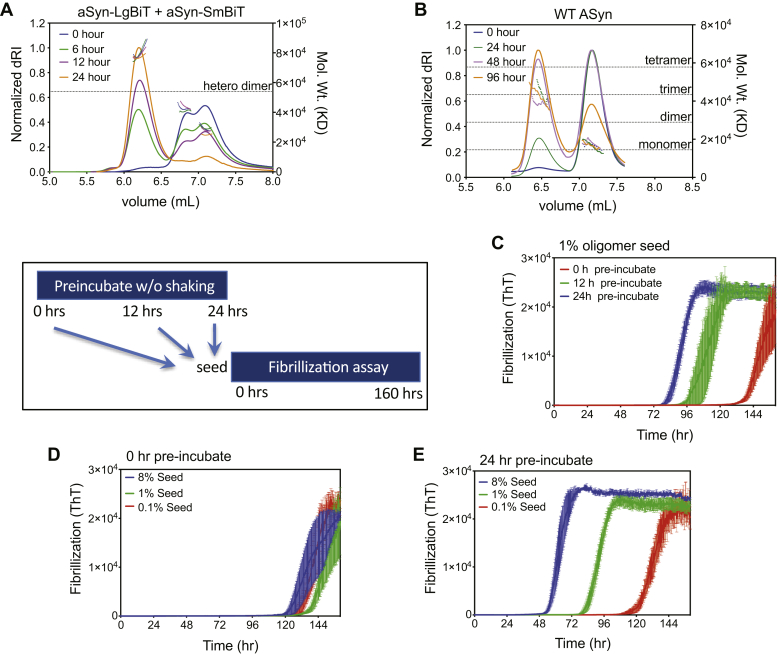
**ASyn-nLuc Oligomers can seed ASyn fibrillization.** In total, 50 μM each of ASyn-LgBiT and ASyn-SmBiT (*A*) or 100 μM untagged ASyn (*B*) was incubated for the indicated times at 37 °C and then analyzed by size-exclusion chromatography with multiangle light scattering (SEC-MALS). Solid lines are refractive index and dotted lines are the calculated molecular weights. ASyn converts from a largely monomeric to a largely trimeric state over the incubation time course of our assays (24 h nLuc, 96 h. FRET). *C*–*E*, preformed split nLuc-tagged ASyn oligomers are used as seeds for an ASyn fibrillization assay as described in methods. nLuc-tagged ASyn oligomers are formed in a still preincubation of ASyn-LgBiT and ASyn-SmBiT and subsequently seeded into an untagged ASyn fibrillization assay. Various percentages of ASyn-nLuc oligomers in a large excess of untagged ASyn are incubated as described in methods and ASyn fibrils formed are quantified using ThT fluorescence. *C*, seeding capabilities increase with preincubation time for a 1% seeding. *D*, zero time of preincubated does not seed fibrillization. *E*, seeding capacity of 24 h preincubated split nLuc-tagged ASyn increases with amount of seed added. Mean ± SEM shown, n = 3.

**Figure 3:**
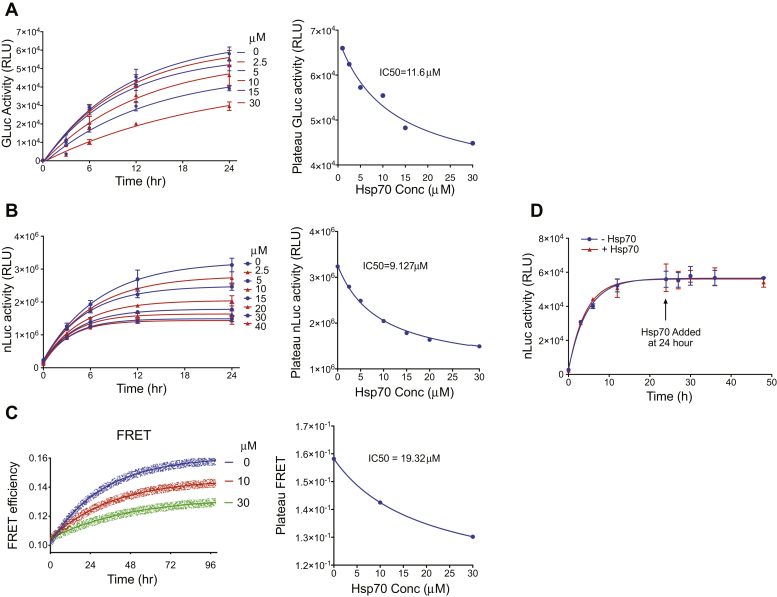
**Hsp70 impairs ASyn oligomerization in multiple assays.** Various concentrations of Hsp70 were added to the (*A*) split gLuc (42), (*B*) split nLuc, and (*C*) FRET (42)-based ASyn oligomerization assays. All assays were as in methods. *B*, in total, 5 μM each of ASyn-SmBiT and ASyn-LgBiT were incubated with 1 mM ADP, 1 mM Mg. *C*, ASyn-Cy3 and ASyn-Cy5 were incubated with 0 (*blue*), 10 (*red*), or 30 (*green*) μM Hsp70 with 1 mM MgCl_2_, 1 mM ADP. Triplicates (*circles*) are shown with first-order kinetic fit (*line*). Hsp70 impaired ASyn oligomerization in all three assays. The IC_50_ of Hsp70 for each assay is calculated based on the plateau signal at 24 h using the GraphPad Prism nonlinear regression model for dose response (three parameters). *D*, in total, 10 μM of each of ASyn-SmBiT and ASyn-LgBiT was incubated for 24 h after which 20 μM Hsp70, 1 mM Mg, and 1 mM ADP were added, and the reactions further incubated. Hsp70 fails to disassemble already formed ASyn oligomers. Means ± s.d. shown, n = 3.

Combined size-exclusion chromatography and multiangle light scattering (SEC-MALS) analyses of either nLuc-tagged (Fig. 2*A*) or untagged ASyn (Fig. 2*B*) species showed that ASyn converts from a largely monomeric to a largely trimeric state over the incubation time course of the assays (24 h for nLuc tagged species, 48 h for untagged FRET assay species (42)), indicating that we are monitoring the early stages of oligomerization. Previously, it was shown that oligomers capable of seeding fibril formation can be formed in *in vitro* biochemical assays (47). To test that our complementation tags do not interfere with fibril seeding ability, we took samples from our nLuc oligomerization assay at different time points and added them to an excess of untagged monomeric ASyn. This mixture was incubated with shaking at 37 °C and fibril formation monitored with ThT fluorescence. The tagged oligomers we had formed in the absence of shaking, which precludes fibril formation (Fig. 1*B*), were able to seed fibril formation in a time- (Fig. 2*C*) and concentration-dependent manner (Fig. 2, *D* and *E*), and thus contained oligomers on pathway for fibril formation. We find that tagged ASyn fibrillizes more readily than untagged ASyn (compare Fig. 1*B* to Fig. 2*D*) and that tagged ASyn oligomerizes more rapidly than untagged species (compare Fig. 3, *A* and *B* to Fig. 3*C*). Tags placed on ASyn are known to accelerate its kinetics of fibrillization but do not significantly alter NMR determined soluble ASyn nor TEM determined amyloid structures (46).

### Hsp70 blocks ASyn oligomerization in an ATP-independent manner

We next tested Hsp70’s impact on ASyn oligomerization. In three ASyn oligomerization assays, split Gaussia luciferase (gLuc) (42) (Fig. 3*A*), nLuc (Fig. 3*B*), and FRET (42) (Fig. 3*C*), Hsp70 impaired oligomer formation at similar IC_50_ concentrations (11.6 μM gLuc, 9.1 μM nLuc, 19.3 μM FRET) and to similar degrees. As the nLuc and gLuc tags bear no homology to each other and the FRET assay does not include tags, direct Hsp70 engagement with the tags is unlikely to play a significant role. In contrast to its ability to block oligomer formation, Hsp70 was unable to disassemble preformed oligomers (Fig. 3*D*), which is consistent with previous findings (24). Because our minimal detectable signal is a mixture of dimer/trimer (Fig. 1, *E* and *F*) and the end reactions contain dimer/trimer (Fig. 2, *A* and *B*), Hsp70 is most likely blocking oligomerization by acting on monomeric ASyn, although a dimer engagement cannot be excluded.

**Figure 4:**
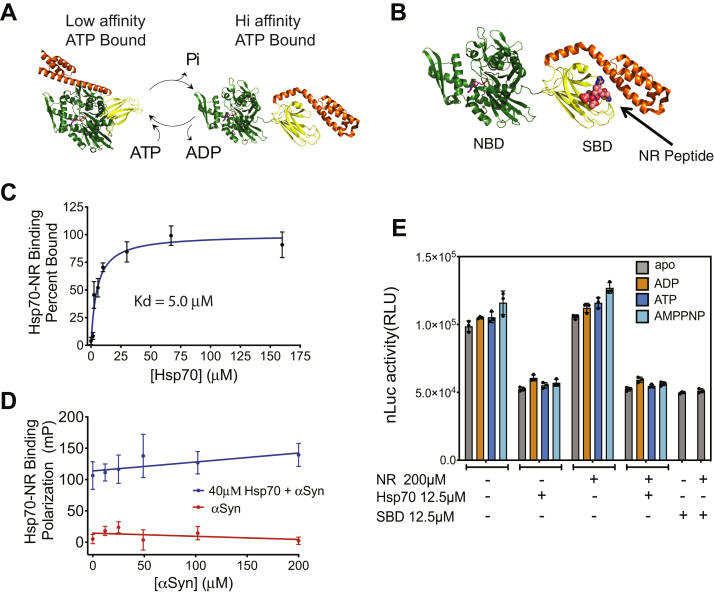
**Hsp70 blockage of ASyn oligomerization requires neither ATP cycling nor binding to the canonical substrate-binding site.***A*, schematic of canonical action of Hsp70 family members. Structures are based on the *E. Coli* Hsp70 homologue, DnaK. ATP hydrolysis drives Hsp70 from a low- (ATP) to a high-affinity (ADP) substrate-binding state. Nucleotides bind to the N-terminal nucleotide-binding domain (NBD, *green*, residues 1–388). Substrate binds to a pocket formed in the C-terminal substrate-binding domain (SBD, *yellow*/*orange* residues (389–602) by the lid region (*orange*, residues 508–602) folding over the β-sheet core of the SBD (*yellow*, residues 389–507) in the ADP-bound conformation. Protein data bank identifiers are 2kho, 4b9q, and 1dkx. *B*, structure of NR peptide bound to the *E. coli* Hsp70 homologue DnaK is shown. NR peptide binds to the canonical substrate-binding site. *C*, binding of NR peptide to Hsp70 was measured by fluorescence polarization of N-terminal 5-FAM (5-Carboxyfluorescein) label on the NR peptide as described in methods. NR binds with a K_d_ of 5.0 μM. Means ± s.d. shown, n = 6. *D*, a competitive binding assay shows that the binding of NR peptide to Hsp70 is not competed by the presence of increasing ASyn (αSyn-WT). Means ± s.d. shown, n = 6. *E*, 10 μM each of ASyn-LgBiT and ASyn-SmBiT, with or without full-length Hsp70 or Hsp70-SBD (see Fig. 5), 2 mM nucleotide and the NR peptide as indicated were incubated in 384-well plates for 24 h and nLuc luciferase activity measured as described in methods. ADP stabilizes the substrate-bound conformation, and AMPNP, a nonhydrolyzable ATP analogue, stabilizes the ATP-bound state. Neither nucleotide, NR nor truncation impacted Hsp70 blockage of ASyn oligomerization. Means ± s.d. shown, n = 3. Lower concentrations of NR peptide likewise had no impact on Hsp70 blockage of ASyn oligomerization (Fig. S6).

It is well appreciated that Hsp/c70s engage substrates *via* a substrate-binding pocket in the SBD. The affinity for substrates at this cleft is regulated by the nucleotide state of the NBD, the ATP-bound state leading to weak affinity, and the ADP-bound state to tight binding (34) (Fig. 4*A*). In the ATP-bound state, the NBD (green in Fig. 4*A*) interacts with both parts of the SBD (yellow and orange in Fig. 4*A*), sequestering the α-helical lid subdomain (orange in Fig. 4*A*) away from the bottom side of the substrate-binding cleft (yellow in Fig. 4*A*) and allosterically lowering affinity even further. In the ADP-bound form, the substrate binds to the canonical substrate-binding pocket formed upon domain rearrangement (Fig. 4, *A* and *B*). Thus, in the canonical model, ATP binding and hydrolysis drive cycles of substrate binding and release (48, 49).

**Figure 5:**
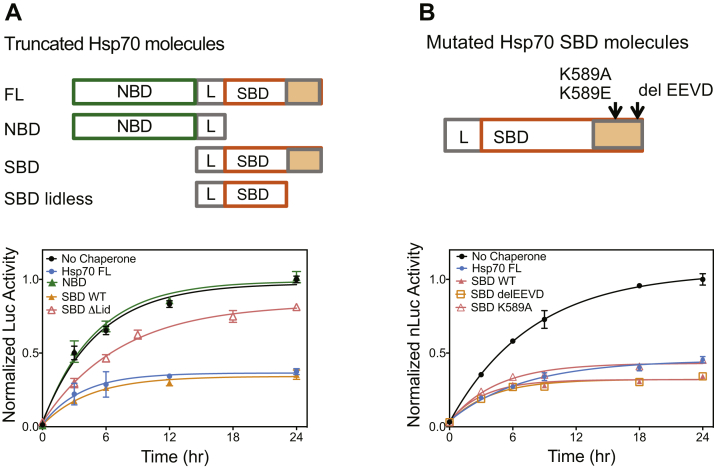
**Activity of truncated and mutated Hsp70 species.***A*, activity of truncated Hsp70 species. *Top*, schematic of Hsp70 truncations. Nucleotide-binding domain (NBD, *green*, residues 1–381), linker region (L, *gray*, residues 382–393), substrate-binding domain (SBD, *orange open* and *filled*, residues 394–641), SBD without the lid region (SBD Δlid, *orange open*, residues 394–510). *Bottom*, Hsp70 fragments are tested for their ability to impair ASyn oligomerization. In total, 10 μM each of ASyn-LgBiT and ASyn-SmBiT was incubated with 10 μM of the indicated Hsp70 and nLuc activity measured as described in methods. Means ± s.d. shown, n = 3. *B*, activity of mutated Hsp70 species. *Top*, schematic of Hsp70 mutations in the Hsp70 SBD. K589A blocks Hsp70 recognition of phosphatidylserine-modified proteins and subsequent engagement for endosomal autophagy. C-terminal EEVD sequence deleted blocks Hsp70’s engagement both with Hsp90, which promotes refolding, and with CHIP, which engages proteasomal degradation. *Bottom*, Hsp70 fragments are tested for their ability to impair ASyn oligomerization. ASyn oligomerization assays were performed as in *A*. Hsp70 impairment of ASyn oligomerization does not depend on Hsp70 interaction sites used for endosomal autophagy nor for Hsp90/CHIP engagement. Means ± s.d. shown, n = 3.

By contrast with this canonical mode of action, previous studies have shown that both Hsp70 and Hsc70 can impair ASyn fibrillization in the absence of ATP cycling and even without the NBD as the SBD alone is sufficient for blockage of ASyn fibrillization (27, 28, 31). To assess whether Hsp70 can block oligomerization in a similar manner, we analyzed the nucleotide dependence of Hsp70’s ability to block ASyn oligomerization in our assay (Fig. 4*C*). We found that neither the addition of ATP nor conditions that blocked ATP cycling (addition of ADP or the nonhydrolyzable ATP analogue, adenosine-5'-[(α,β)-imido]diphosphate (AMPPNP)) impacted Hsp70’s ability to block oligomerization (Fig. 4*C*). As was shown for Hsp70 blockage of ASyn fibrillization (27, 28, 31), the NBD was also not required to block oligomerization, as the SBD alone had activity equivalent to the full-length Hsp70 (Figs. 4*C* and 5A). Taken together, these data are consistent with a holdase model of Hsp70 action, in which the Hsp70 SBD is able to sequester ASyn monomers, blocking their ability to oligomerize or form fibrils, without input from the NBD.

### Hsp70 does not engage ASyn *via* its canonical substrate-binding site

It has generally been assumed that Hsp70 engages ASyn *via* its canonical substrate-binding site. To determine if this was the case, we tested the ability of a well-characterized Hsp70 substrate, the NR peptide, to compete with ASyn binding, thereby restoring the ability of ASyn to oligomerize. As determined using a fluorescent polarization assay, the NR peptide binds Hsp70 with a Kd of 5.0 μM (Fig. 4*C*) yet ASyn is incapable of competing with NR-Hsp70 binding even at concentrations up to 200 μM (Fig. 4*D*), indicating that ASyn is not engaging Hsp70’s canonical substrate site. Strikingly, NR peptide does not inhibit Hsp70’s action on ASyn oligomerization at concentrations up to 200 μM (Fig. 4*E*). This result was unexpected, and it indicates that the part of the SBD required to block ASyn oligomerization is distinct from the canonical substrate-binding pocket. Further, the NR peptide had no impact on the activity of full-length Hsp70 regardless of nucleotide state (Fig. 4*E*), indicating that the noncanonical ASyn-binding site must be fully available independent of Hsp70 domain organization or of occupancy of the canonical substrate-binding pocket.

To define what part of the SBD was most relevant for inhibiting ASyn oligomerization, we examined the activity of additional Hsp70 truncations. We removed parts of Hsp70 that are known to interact with cochaperones or engage in other PPIs. Specifically, cochaperones are known to bind the linker between the NBD-SBD and to EEVD residues at the extreme C-terminus of the lid domain (41). We found that a version of Hsp70’s NBD, which includes the interdomain linker, was still inactive (Fig. 5*A*), suggesting that this PPI site was not involved. The EEVD sequence mediates Hsp70 engagement both with Hsp90, which promotes refolding, and with the C terminus of Hsc70-interacting protein (CHIP), which promotes proteasomal degradation (41). Deleting the C-terminal EEVD residues did not alter the ability to block ASyn oligomerization *in vitro* (delEEVD Fig. 5*B*), showing that this known PPI site is also not responsible. However, we found that deleting the entire lid partially impaired the activity of the SBD (Fig. 5*A*), suggesting that at least part of this subdomain, outside the EEVD, could play a role. This partial dependence on the lid is consistent with prior results (27, 31) showing that deletion of the lid region partially impairs Hsp/c70s blockage of ASyn fibrillization. Finally, we introduced a point mutant, K589A, which is sufficient to block binding of Hsp70 to endosomes (41, 50), but found that it too was not relevant for blocking ASyn oligomerization (K589A, Fig. 5*B*). Together these results suggest that a unique and as yet unmapped surface on the lid domain and the core β-sheet domain are required for productive engagement of Hsp70 with ASyn.

### A similar Hsp70 mechanism blocks ASyn oligomerization in cells

We next extended these biochemical observations to H4 neuroglioma cells to understand if a similar Hsp70-ASyn interaction was relevant in cells. We found that the previously reported cell model using overexpression of split gLuc tags on ASyn (19) drives ASyn into large S129 phospho-synuclein positive aggregates in H4 cells (Supporting information and Fig. S1). We anticipated that the presence of these large aggregates could complicate analyses of oligomerization using the split gLuc tagged system, so we established an alternative, quantitative cellular ASyn oligomerization assay using the split nLuc complementation system, which does not form such aggregates (Fig. S1). We established conditions for both cell handling and assay parameters in which nLuc activity resulting from split nLuc tag complementation was quantitatively proportional to the amount of tagged ASyn transiently overexpressed for both cellular and media compartments (Supporting information and Fig. S2). This assay provided an alternative cell-based platform for studying the interaction of Hsp70 with ASyn.

**Figure 6:**
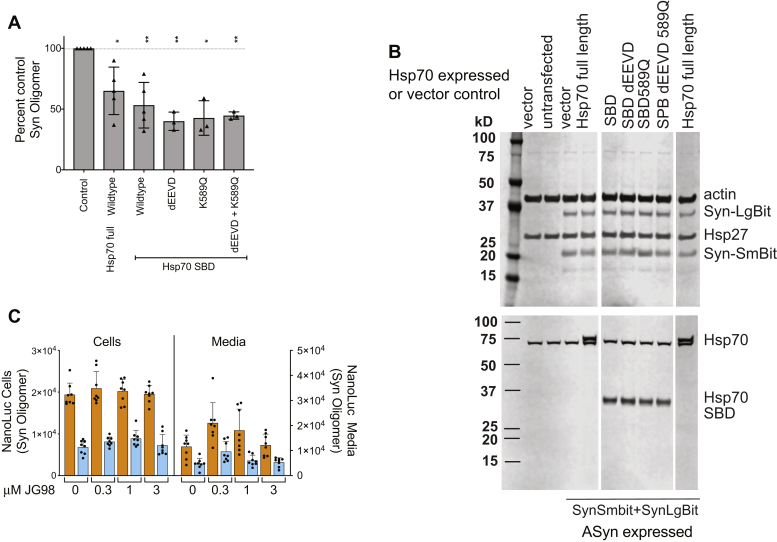
**Neither ATPase activity nor downstream engagements are required for Hsp70-mediated inhibition of oligomerization in H4 neuroglioma cells.***A*, impact of overexpression of full-length and truncated Hsp70 without or with mutations blocking downstream pathways on ASyn oligomer formation in H4 cells transiently transfected with vectors coexpressing ASyn-LgBiT and ASyn-SmBiT. Samples normalized to split NanoBit-tagged ASyn (ASyn-SmBit+ASyn-LgBit) control without Hsp70 are compiled from multiple experiments. Each symbol is the mean of 6 to 12 wells of a separate experiment and error bars are shown ±s.d. Statistics are one sample *t*-test two-tailed for difference from control value of 100, ∗*p* < 0.05, ∗∗ < 0.01. All Hsp70 variants tested impaired ASyn oligomer formation in H4 cells. *B*, western analyses of transfected and endogenous proteins from samples run in parallel to panel *A*. Expression of transfected proteins is robust and consistent and does not impact levels of actin, endogenous Hsp70 nor the stress protein Hsp27, indicating that overexpression of mutant Hsp70 does not perturb cellular proteostasis. *C*, vector (*orange*) or full-length Hsp70 (*cyan*) is cotransfected with vectors expressing ASyn-LgBiT or ASyn-SmBiT and treated with 0.1% DMSO alone or containing allosteric Hsp70 ATPase inhibitor JG98 at nontoxic doses that are ≥3-fold higher than the previously reported EC_50_s (55). JG-98 was toxic at 10 μM. Fugene transfection was as described in Supporting information. ASyn oligomers were measured by assaying H4 cells and media at 48 and 24 h, respectively, post transfection for reconstituted nLuc activity. Hsp70 ATPase inhibition by JG98 had no impact on its ability to block ASyn oligomerization in cells. Mean ± s.d. with individual wells shown, n = 8.

Using this assay, we found that overexpression of either full-length Hsp70 or the Hsp70 SBD reduced ASyn oligomers in H4 neuroglioma cells (Fig. 6*A*). Reproducible expression of transfected ASyn-LgBit and of ASyn-SmBit in each of the samples was confirmed by western blot analyses (Fig. 6*B*). Neither the wildtype nor mutant SBD constructs used here showed any indication of toxicity or perturbation of proteostasis, as evidenced by the unchanged cell numbers (Fig. S3) and the consistent levels of actin and markers of the stress responses (Hsp27 and endogenous Hsp70) (Fig. 6*B*). The full activity of the SBD in blocking oligomerization in H4 cells indicates that an ATP-independent mode of Hsp70 action can block ASyn oligomerization in these cells.

Unexpectedly, the transfected Hsp70 runs slightly higher than the endogenous human H4 cell Hsp70. The smaller endogenous band is unlikely to result from cross-reaction with Hsc70 or binding immunoglobulin protein (BiP), as these Hsp70 homologues are both larger than Hsp70. Rather, this effect could be due to expression of a different human Hsp70 variant in the H4 neuroglioma cell line. Indeed, while the sequence we chose to overexpress was based on the consensus entries for HSPA1A (GenBank NM_005345.6 and BC002453.2), GenBank also lists an alternative HSPA1A form (M11717.1), in which two amino acids are changed and one deleted. A more likely possibility is that H4 neuroglioma cells express the Hsp70 variant HSPA2, which is two amino acids smaller than the HSPA1A version we overexpressed (51).

We confirmed the ATP independence of Hsp70 blocking ASyn oligomerization in H4 cells using ATPase inhibition. JG-98, an allosteric Hsp70 ATPase inhibitor that impairs Hsp70’s canonical action in other pathways (52, 53, 54, 55), did not block Hsp70’s ability to reduce ASyn oligomers in media or cellular compartments of H4 cells transfected with either control or Hsp70-expressing vector (Fig. 6*C*). As in our biochemical analyses, mutations preventing engagement with known PPIs, such as the EEVD deletion and the endosome blocking K589Q mutation (41, 50), did not block the ability of the SBD to reduce ASyn oligomer levels in cells (Fig. 6*A*). As was the case for the SBD, none of these mutations showed toxicity or perturbation of proteostasis (Fig. 6*B* and Fig. S3). Thus, in both our cellular and biochemical assays, Hsp70 does not act to block ASyn oligomerization through an ATPase-dependent mechanism nor *via* previously identified, noncanonical SBD binding sites. Together our biochemical and cellular data indicate that this important interaction occurs through a new noncanonical site, which involves parts of the SBD and lid.

## Discussion

Although Hsp70 protects against ASyn pathogenicity in cell and animal models (17, 18, 19, 20, 21, 22), little is known about how it exerts this effect. Biochemical investigations demonstrate that Hsp/c70s block ASyn fibrillization (24, 26, 27, 28, 29, 30, 31, 32, 33) *via* an ATP-independent holdase mechanism (27, 28, 29, 30, 31, 32). But because fibrillization is a complex and variable multistep process, the stage at which this inhibition occurs is not clear. In particular, Hsp70’s impact on ASyn oligomerization, the initial step of fibrillization, has not been directly determined (26, 27, 28, 30, 31). Using both previously reported assays (42) and a novel, biochemical ASyn oligomerization approach, we show that Hsp70 directly blocks early-stage ASyn oligomerization in a nucleotide-independent manner. Surprisingly, we show that a competitive inhibitor of the Hsp70 canonical substrate-binding site, the NR peptide, does not block this action nor does ASyn block NR binding, indicating that a noncanonical site must be responsible for retarding ASyn oligomerization. Thus, it may be possible to separate Hsp70’s blocking of ASyn oligomerization from its action on other necessary cellular substrates.

Previous studies investigating the impact of Hsp/c70s on ASyn misfolding have used ASyn fibrillization assays (26, 27, 28, 29, 30, 31, 32, 33). Engagement of Hsp/c70s with ASyn fibrils has complicated these analyses. ATP-dependent binding of Hsp/c70s to ASyn fibrils aggregates Hsp70 (29), competes effectively with chaperone engagement with soluble ASyn (32), and impairs Hsp/c70s blockage of fibrillization (30, 32). Our ability to follow ASyn oligomer formation under conditions in which subsequent higher-order prefibrils and fibrils are not formed has allowed us to cleanly investigate the mechanism by which Hsp70 prevents early-stage ASyn oligomerization. Despite the challenges inherent in fibrillization studies, many of their conclusions are consistent with our data. Hsp/c70s block fibrillization by acting on a prefibrillar, soluble form of ASyn (26, 27, 28, 30, 31, 32, 33, 56) in an ATP-independent mode (27, 28, 29, 30, 31, 32), with full activity from the SBD alone (27, 28, 31) and partial activity upon lid removal (27, 31). The soluble prefibrillar ASyn species identified by prior studies as the target of Hsp70 engagement could be high-order oligomers as at least 15-mers are needed before adopting beta-sheet structure (57) and larger species could be seeding fibrillization. Our data indicate that Hsp70 is most likely acting on monomeric or possibly dimeric ASyn to block oligomerization. The similarities of Hsp70 action blocking fibrillization with its blocking oligomers suggest this is the mechanism by which Hsp/c70s block ASyn fibrillization as well. A number of studies indicate that Hsp70 binds monomeric ASyn (28, 29, 32, 56) supporting such engagement.

Recently, a few examples of Hsp70 interactions distinct from the canonical substrate-binding site have been found to drive Hsp70 engagement with XIAP substrates (58) and downstream autophagic and cochaperone actions (41). As the XIAP substrates bind to the NBD of Hsp70 (58) and ASyn oligomerization blocking activity lies within the SBD, they cannot share an Hsp70 engagement site. Deletion of the C-terminal EEVD motif of Hsp70, which engages Hsp90 to coordinate protein refolding as well as CHIP to mediate transfer of proteins to the proteasome (41), had no impact on Hsp70’s ability to block ASyn oligomerization. The Hsp70 mutation K589Q, which blocks Hsp70 directed transfer of phosphatidyl serine modified proteins to endosomal autophagy (41, 50), also had no impact on ASyn oligomerization. Thus Hsp70’s ability to suppress ASyn oligomerization relies on a previously unidentified noncanonical interaction site. Interestingly, the Hsp70 region responsible for chaperone-mediated autophagy is as yet unidentified, so could not be directly tested and remains a possible mediator of this interaction. The requirement of both the lid and the remainder of the SBD for full activity is consistent with cross-linking studies, which show ASyn binding to both SBD domains near the canonical site (56). Higher-resolution structural studies will be needed to definitively determine the binding site of ASyn on Hsp/c70s.

To probe the mechanism of Hsp70 suppression of ASyn oligomerization in H4 neuroglioma cells, we established a new cellular ASyn oligomerization assay based on complementation of split nLuc tags placed on ASyn. In our hands, split gLuc tags drive ASyn into large aggregates, possibly confounding analyses of oligomerization. The split nLuc-tagged system has the advantage of very low intrinsic binding of the split tags and furthermore did not drive ASyn aggregation. Using this novel assay, we found that Hsp70 suppression of ASyn oligomerization is mediated by an ATP-independent mechanism, as it is driven entirely by the SBD and is insensitive to Hsp70 ATPase inhibition. Furthermore, as was the case in our biochemical experiments, it does not rely upon previously mapped noncanonical Hsp70 engagement regions. Thus, a similar mechanism of Hsp70 suppression of ASyn oligomerization is at play in cells and in our *in vitro* assay.

**Figure 7:**
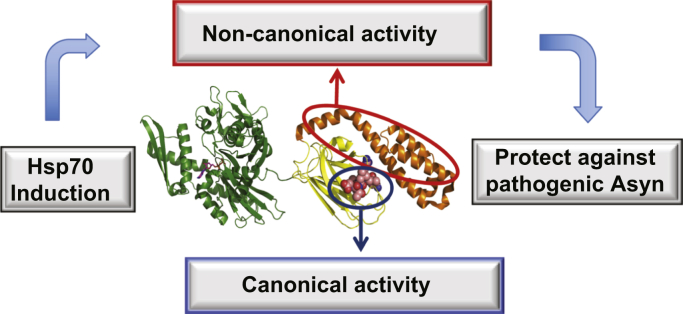
**Hypothesis: Novel engagement of Hsp70 with ASyn *via* a site separate from the canonical substrate-binding site leads to specific and protective action on ASyn.** We propose a novel mechanism in which a noncanonical alternative binding site for ASyn on Hsp70 blocks ASyn oligomerization and confers protection against ASyn pathogenicity.

Because ASyn oligomers are likely the most pathogenic species of misfolded ASyn (5, 59), we hypothesize that the novel noncanonical Hsp70 site responsible for blocking ASyn oligomerization is protective against ASyn pathogenicity in animal models and in disease (Fig. 7). Further biochemical and structural mechanistic studies will be needed to determine the exact molecular mechanisms of Hsp70 suppression of ASyn oligomerization. Such studies will enable targeted mutagenesis of Hsp70 ASyn engagement sites to be tested *in vivo* for protection against neurodegeneration to directly test our hypothesis.

The discovery that Hsp70 blocks ASyn oligomerization in a novel manner makes it possible to develop therapeutic approaches that enhance these beneficial effects without interfering with Hsp70’s many cellular functions. An underlying assumption of the field has been that protective Hsp70 actions on ASyn all occur *via* its canonical substrate-binding site. An important and mistaken inference from this assumption is that a chemical modulator of Hsp70’s ATP cycling or canonical interactions would influence all of Hsp70’s many roles in the cell. This scenario will likely result in untoward effects complicating therapeutic application for neurodegenerative diseases. For example, activation of Hsp70 canonical actions increases epileptic activity *via* impact on potassium currents (60). Such activation would be undesirable in Alzheimer’s disease and LBD, which show aberrant network hyperactivity associated with cognitive abnormalities (61, 62). An alternative therapeutic approach would be to target Hsp70’s noncanonical action on ASyn with ASyn pharmacological chaperones (42, 63), small molecules that bind to and stabilize select ASyn conformations. Such compounds could impact ASyn’s mode of interaction with Hsp70, allowing for enhanced blocking of ASyn oligomer formation. In support of this proposed approach, we have reported the development of small-molecule pharmacological chaperones that engage ASyn and in so doing reverse multiple different ASyn malfunctions (42, 64). Alternatively, it is possible that allosteric modulating sites exist on Hsp70 whose engagement by small molecules could enhance this desirable activity. Hsp70 is a complex molecule with significant allosteric modulation by cofactors. Small-molecules activators of canonical Hsp70 activity have been identified (65). Given Hsp70’s structural complexity and rich opportunity for regulatory pockets, it is possible that such activating molecules can be found for noncanonical Hsp70-ASyn engagement in the SBD lid region as well.

In conclusion, these studies indicate that Hsp70 engages ASyn to prevent oligomer formation in a novel manner that is distinct from its normal canonical substrate-binding site. This finding implies that specifically targeting Hsp70 to ASyn for treatment of PD, LBD, and related synucleinopathies is possible. A high priority will be to determine the exact molecular mechanism of this novel activity.

## Experimental procedures

Additional methods are available in Supporting information.

### Molecular cloning

#### Bacterial expression vectors

Gaussia luciferase (pET28b-αSyn-GLuc1 and pET28b-αSyn-GLuc2), αSyn-WT (pET28b-αSyn), and FRET (pET28b-aSyn-Q99C) vectors were generated as described (42). NanoBit luciferase (nLuc) was split into two fragments: LgBiT (N-terminal 158 residues); SmBiT (C-terminal 11 residues) (43). Either LgBiT or SmBiT was fused to the C terminus of human ASyn with a flexible linker (GGGGSGGGGSSG) placed between ASyn and the nLuc tags. The bacterial expression vectors pET28b-αSyn-LgBiT and pET28b-αSyn-SmBiT were constructed by inserting either coding region into pET28b vector (Novagen) *via Nco*I/*Not*I sites in the multiple cloning site. The human Hsp70 coding region (HSPA1A, Genecopia, amino acid sequence identical to GenBank: BC002453.2, Uniprot: P0DMV8) was amplified using Q5 High-Fidelity DNA Polymerase (New England Biolabs) and inserted into the TOPO cloning site in pET151/D-TOPO *E. Coli* expression vector (Invitrogen). The pET151/D-TOPO-Hsp70 plasmid was used for recombinant expression of Hsp70 protein. Truncated Hsp70 constructs were made using a Q5 site-directed mutagenesis kit (New England Biolabs) according to the manufacturer’s instructions. YDJ1 was expressed in the pET151 bacterial expression plasmid with a cleavable 6× His-tag. All plasmids were verified by sequencing.

#### Mammalian expression vectors

pMKHsp70-1A: The human Hsp70 coding region (HSPA1A, GenBank: BC002453.2, and NM_005345) from the 2072 bp *Nhe*I-*Xba*I fragment of pcDNA3.1-Hsp70 (gift from Dr Chad Dickey) was combined with the 7035 bp *Xba*I-*Nhe*I fragment of expression vector pMK1252 (gift from Dr Martin Kampman, UCSF). pVLHsp70-1A: The human Hsp70 coding region (HSPA1A, GenBank: BC002453.2, and NM_005345.6) from the 1991 bp *Bam*HI-*Eco*RI(filled in) fragment of pcDNA3.1-Hsp70 was cloned into the *Bam*HI/*Hpa*I polylinker sites of pLV-Bobi (66). The inserted fragment and flanking vector regions for all constructs were sequenced and verified using at least four independent runs covering both strands. pLV-Bobi (66), an empty expression vector, and pLV-αSyn vector (LV-αSyn) (67) expressing untagged wildtype human ASyn were gifts from Dr Brian Spencer of UCSD.

### *E. coli* protein expression and purification

BL21(DE3) *Escherichia coli* cells were transformed with desired plasmids and cultured in LB media at 37 °C. At OD_600_ 0.6 to 0.8, the culture temperature was lowered to 20 °C, IPTG (GoldBio) was added at 0.5 mM to induce protein expression and cultures further incubated at 20 °C overnight. The cells were harvested by centrifugation at 4000*g* for 20 min in an Avanti J-26 XPI centrifuge with a JLA 8.1000 rotor (Beckman Coulter).

#### Proteins αSyn-GLuc1, αSyn-GLuc2, αSyn-LgBiT, Hsp70, YDJ1

Plasmid-transformed *E. coli* cells were resuspended in 25 mM Tris, 500 mM NaCl, 0.5 mM TCEP, pH 8.0 and then lysed in the presence of EDTA-free Protease Inhibitor Cocktail (Roche Life Sciences) using an EmulsiFlex-C3 (Avestin). The lysate was cleared by centrifugation at 30,000*g* for 30 min in a JA 25.50 rotor (Beckman Coulter). The His-tagged target protein was purified by Ni-NTA gravity-flow chromatography (Qiagen). The eluted protein was subjected to MonoQ 10/100 GL (GE Healthcare Lifesciences) chromatography using a 0 to 600 mM NaCl gradient elution. αSyn-GLuc1 and αSyn-GLuc2 were polished by HiLoad 16/60 Superdex 75 size-exclusion chromatography (SEC) (GE Healthcare Lifesciences), collecting fractions eluting at 47 to 61 ml. αSyn-LgBiT and Hsp70 were polished by HiLoad 16/60 Superdex 200 SEC (GE Healthcare Lifesciences); fractions eluting at 68 to 82 ml (αSyn-LgBiT) and 78 to 86 ml (Hsp70) were collected. The harvested protein was concentrated by Amicon Ultra-15 centrifugal filter units (Millipore Sigma), filtered through a 0.22 μm filter (E&K Scientific), flash frozen, and stored in aliquots at −80 °C. YDJ1 was purified as previously described (68).

#### Proteins αSyn-SmBiT, αSyn-Q99C

Plasmid transformed *E. coli* cells were resuspended in 20 mM Tris, pH 8.0 and lysed by boiling for 30 min in the presence of an EDTA-free Protease Inhibitor Cocktail (Roche Life Sciences). The lysate was cleared by centrifugation at 30,000*g* for 30 min in a JA 25.50 rotor (Beckman Coulter). Streptomycin sulfate was added to the lysate at 10 mg/ml to precipitate DNA. After a 30-min incubation at 4 °C, the lysate was cleared by centrifugation at 30,000*g* for 30 min. Ammonium sulfate was added to the lysate at 0.36 g/ml to precipitate protein. After incubation at 4 °C overnight, the protein was pelleted by centrifugation at 30,000*g* for 30 min. The protein was resuspended in 20 mM Tris, 1 mM DTT, pH 8.0, then subjected to MonoQ 10/100 GL (GE Healthcare Lifesciences) chromatography using a 0 to 600 mM NaCl gradient elution. αSyn-SmBiT and αSyn-Q99C were polished by HiLoad 16/60 Superdex 200 SEC (GE Healthcare Lifesciences), collecting fractions eluted at 78 to 86 ml. The harvested protein was concentrated, filtered, and stored as described above.

#### Protein αSyn-WT

Untagged wild-type ASyn (αSyn-WT) purification was performed by periplasmic lysis as previously described (69). ASyn was eluted at 350 to 370 mM salt by ion-exchange chromatography (MonoQ 10/100 GL—GE Healthcare Lifesciences). Fractions containing pure ASyn as determined by SDS-PAGE were dialyzed against PBS overnight at 4 °C. Dialyzed ASyn was aliquoted, flash frozen in liquid N_2_, and stored at −80 °C. For each experiment, ASyn fresh stock solution was rapid thawed, filtered through a 0.22 μm filter (Millipore), and further purified by gel filtration chromatography (Superdex 200 Increase 3.2/300, Ge Healthcare) to obtain monomeric fractions.

### Biochemical ASyn oligomerization assays

The split Gaussia luciferase (gLuc)-based oligomerization assay was performed as described (42) (Supporting information) and used for Figure 3*A*, with the exception that 1 mM ADP, 1 mM MgCl_2_ and Hsp70 at various concentrations were added at the beginning of the assay. The Förster resonance energy transfer (FRET) oligomerization assay was carried out as described (42) (Supporting information), except that 1 mM ADP, 1 mM MgCl_2_, and Hsp70 at various concentrations were added in Figure 3*C*. For the split NanoLuc luciferase (nLuc) oligomerization assay, different concentrations of αSyn-LgBiT and αSyn-SmBiT were mixed at a 1:1 M ratio in PBS (pH 7.4). The mixtures were incubated at 37 °C in micro PCR tubes on a thermal cycler (BioRad DNA Engine PTC-200). At the indicated time points, 10 μl of the mixture was placed in a single well of a black/clear bottom 384-well microplate (Greiner). Oligomerization was quantified by measuring the luminescence on a SpectraMax L Microplate Reader (Molecular Devices) using either an h-CTZ protocol or a NanoFuel protocol. For the h-CTZ protocol, 40 μl of h-CTZ (40 μM, NanoLight Technology) was injected into the wells with the samples using the injector integrated in the SpectraMax L. The luminescence was measured with 2-s delay and 3-s integration times. The h-CTZ protocol applies to Figures 1, *A*, *C* and *D*, 3, *B* and *D*, 4C, 5, *A* and *B*. Alternatively, for the Nanofuel protocol, NanoFuel GLOW Assay kit (NanoLight Technology) reagent was added into each well according to manufacturer’s instructions. The luminescence measurements were performed on the SpectraMax L with 8-min delay and 1-s integration times. This protocol was used for Figures 1*B* and 4C. In experiments used to evaluate Hsp70 impact, 1 mM nucleotide (ATP, ADP, or AMPNP) and 1 mM MgCl_2_ and Hsp70 or Hsp70 mutants at different concentrations were added as shown at the indicated time points (Figs. 3, *B* and *D*, 4C, 5, *A* and *B*, Fig. S5). For all three assays 1 μM YDJ1, an hsp40, which stimulates ATP cycling and presents clients to Hsp70, was added in some cases (Fig. 3, *A*–*D*) until it was found to have no impact (Fig. S5).

### ASyn fibrillization assays

To measure fibrillization, ASyn proteins were incubated with or without shaking at 1000 rpm on a ThermoMixer (Eppendorf 5350) at 37 °C as indicated. The dye Thioflavin T (ThT) is widely used to detect amyloid fibrillization as it fluoresces upon binding to fibrils (70). Samples were diluted 20-fold into 25 μM Thioflavin in PBS and incubated at room temperature for 15 min to 1 h in 384 plates. ThT fluorescence was read on a SpectraMax M5 microplate reader (excitation 440 nm, emission 482 nm) in endpoint mode. This protocol applies to Figure 1*B*. For experiments measuring seeding of ASyn fibrillization (Fig. 2, *C*–*E*), ASyn-Nluc oligomers were formed by incubating 10 μM each of ASyn-LgBiT and ASyn-SmBiT for 0, 12, or 24 h at 37 °C in a thermal cycler (BioRad DNA Engine PTC-200). The preformed oligomers were used as seeds for an ASyn fibrillization assay. Untagged monomeric ASyn was seeded with 8%, 1%, or 0.1% (molar ratio) of the seeds to a final ASyn protein concentration of 200 μM and incubated at 37 °C with shaking in 384-well microplate with clear bottom (Greiner) sealed with AlumaSeal II (Hampton Research) to prevent evaporation. ASyn fibril formation was quantified by reading ThT fluorescence on a SpectraMax M5 microplate reader (excitation 440 nm, emission 482 nm) in kinetic mode over the time course of fibrillization.

### Size-exclusion chromatography coupled to multiangle light-scattering analysis (SEC-MALS)

For αSyn-LgBiT/αSyn-SmBiT oligomers, recombinantly expressed and purified αSyn-LgBiT and αSyn-SmBiT were mixed at a 1:1 M ratio in PBS to a final concentration of 50 μM each. The mixture was incubated at 37 °C in PCR tubes on a thermal cycler (BioRad DNA Engine PTC-200). After various times, 50 μl of the mixture was taken and subject to SEC using a Shodex KW-802.5 column (HICHROM) pre-equilibrated in PBS. The SEC was coupled to a static 18-angle light-scattering detector (DAWN HELEOS-II, Wyatt Technology), a UV detector, and a refractive index detector (Optilab T-rEX, Wyatt Technology). Data collection was done at a flow rate of 0.35 ml/min. Data was processed using the analysis package as part of the program ASTRA, yielding the reported molar masses of the protein complexes. For untagged ASyn oligomers, recombinantly expressed and purified untagged ASyn was diluted to 100 μM in PBS. The protein was incubated at 37 °C in PCR tubes on a thermal cycler. At 0, 24, 48, or 96 h post incubation, 50 μl of samples was taken and analyzed by SEC-MALS. The data collection and processing were carried out following the same protocol for αSyn-LgBiT/αSyn-SmBiT.

### Binding of NR peptide to Hsp70

NR peptide was synthesized with an N-terminal 5-FAM (5-Carboxyfluorescein) modification by GenScript with purity 98.7%. Hsp70 was titrated while the NR-peptide concentration remained constant at 20 nM. For ASyn titration, Hsp70 and NR concentration remained constant at 40 μM and 20 nM respectively. Experiments were performed in sextuplicate, in filtered PBS with 1 mM MgCl_2_ and 1 mM ADP. Protein concentration of Hsp70 and ASyn was verified with NanoPhotometer NP80 (Implen), using a calculated extinction coefficient at 280 nm of $\varepsilon_{280}^{0.1\%}$ 0.477 and 0.354 respectively (ProtParam – ExPASy (71)). Fluorescence polarization of the NR peptide was measured on a SpectraMax M5 plate reader (Molecular Devices) with excitation and emission wavelengths of 485 nm and 538 nm respectively at 24 °C. Binding curves were plotted using Prism 8.4.3 and fit with a nonlinear regression single site-binding equation curve to obtain a K_d_.

### Western analyses

Western analyses were run as described (42) with the following changes: Purified mouse anti-ASyn antibody clone 42 (BD Biosciences) was diluted at 1/1000, rabbit anti-Hsp70 antibody (Enzo Life Science, Inc, ADI-SPA-812) and mouse monoclonal antibody G3.1 anti Hsp27 antibody (Enzo Life Science, Inc) were diluted at 1/1000, and mouse anti-actin antibody clone AC15, (Sigma-Aldrich) was diluted at 1/35,000. Membranes were then washed four times for 10 min each in PBS 0.1% Tween (PBST) and incubated with secondary antibody donkey anti-mouse infrared 680 and goat anti-rabbit infrared 800 (LI-COR) diluted at 1/10,000 in Odyssey PBS Blocking Buffer (LI-COR, Inc) with 0.2% Tween 20 followed by washing four times for 10 min each in PBST. Membranes were scanned and quantitated using the Odyssey CLx Imaging System (LI-COR). Hsp70 was visualized in the 800 nm fluorescent channel and actin, αSyn, Hsp27, and the molecular weight markers visualized at 700 nm. As previously described (42), proteins are cross-linked to blots by 30 min treatment with 0.4% PFA in PBS prior to staining to enhance ASyn binding (72) to ensure visualization of all ASyn.

### Cellular nLuc assays

H4 neuroglioma cells (HTB-148; ATCC) were passaged in DME containing 10% fetal calf serum (FCS). The cells were plated into polyD-Lysine-coated clear-bottom white well 96-well plates at 7 × 10^3^ cells per well for the oligomerization assays and into 6-well plates at 1.5 × 10^5^ cells per well for western blotting. The following day, cells were transfected using either JetPrime (Polyplus) or Fugene (Promega) according to the manufacturer’s directions. For Figure 6, *A* and *B* DNA and JetPrime were used at a ratio of reagent to DNA of 2:1. Plasmids ASyn-Smbit to ASyn-Lgbit at a ratio of 6:1 were mixed with either Hsp70-expressing vectors or with control vector not expressing protein (pLV-Bobi) with ratio of ASyn plasmids to Hsp70 expressing or control plasmid of 3:7. After 4 h, the DNA-containing media was removed and cells were fed fresh DME media containing 10% FCS. The following day, the media was changed to Opti-MEM without phenol red (Opti-MEM) with penicillin and streptomycin in the media. The next day 80 μl of cellular media was transferred into a 96-well white plate for luciferase measurements. To control for possible impacts of protein overexpression on cell numbers, the cells were incubated with Opti-MEM containing the DNA binding dye Hoechst 33342 (Invitrogen) diluted at 1/5000 for 30 min. The plates were read before and after adding Hoescht on a microplate reader SpectraMax M5 (Molecular Devices) with an excitation and emission wavelengths of 350 nm and 490 nm respectively. The media was removed and replaced with 100 μl of Opti-MEM and cells assayed for luciferase activity. Fugene (Fig. 6*C*) transfection was carried out with a 4:1 ratio of reagent to DNA. Twenty-four hours after transfection the cellular media was replaced with Opti-MEM containing 0.1% DMSO alone or with drugs and incubated a further 24 h. In total, 80 μl of cellular media was removed for luciferase activity measures, the cells were washed in PBS and 100 μl of Opti-MEM placed in the well, and cells assayed for luciferase activity.

Luciferase activity was quantified by measuring the luminescence on a SpectraMax L Microplate Reader (Molecular Devices). Briefly, 80 μl for media or 100 μl for cells of h-CTZ (40 μM, NanoLight Technology) in Opti-Mem was injected into the wells using the injector integrated in the SpectraMax L. The luminescence was measured with 15-s delay and 5-s integration times. The impact of compounds on H4 cell toxicity was assayed using the CytoTox-Glo kit (Promega).

The synthesis and characterization of compound JG-98 were previously described (55). The impact of compounds on H4 cell toxicity was assayed using the CytoTox-Glo kit (Promega).

### Statistical analysis methods

Statistical analyses were run using GraphPad Prism software as described. Multiple samples were compared using one-way ANOVA with Dunnett’s and an alpha of 0.05. In Figure 6, one sample *t*-test two-tailed for difference from control (100) was used. General practice significance nomenclature was used (0.1234 (ns), 0.0332 (∗), 0.0021 (∗∗), 0.0002 (∗∗∗), <0.0001 (∗∗∗∗) unless otherwise indicated).

### Laboratory health and safety procedures

All mandatory laboratory health and safety procedures have been complied with in the course of conducting the experimental work reported herein.

## Data availability

All data are contained in the article and Supporting information.

## Supporting information

This article contains supporting information.

## Conflict of interest

D. A. A. and L. M. are inventors on patent applications covering the assays described herein.
