# Supplementary material for: Hsp70 chaperone blocks α-synuclein oligomer formation via a novel engagement mechanism
Source: J Biol Chem. 2021 Mar 30;296:100613. doi: 10.1016/j.jbc.2021.100613 (PMC8102405; doi:10.1016/j.jbc.2021.100613)
Supplement: Supporting information and Figures S1–S6 [file mmc1.pdf]

Supporting Information

Title: Hsp70 chaperone blocks α-synuclein oligomer formation via a novel engagement mechanism

Authors
Jiahui Tao[1, *], Amandine Berthet[2, *], Y. Rose Citron[1], Paraskevi L. Tsiolaki[1], Robert Stanley[2], Jason E. Gestwicki[3], David A. Agard[✉1, *], and Lisa McConlogue[✉1,2, *]
[1] Department of Biochemistry and Biophysics, University of California San Francisco, San Francisco, CA 94143, USA
[2] Gladstone Institute of Neurological Disease, San Francisco, CA 94158, USA
[3] Department of Pharmaceutical Chemistry, Institute for Neurodegenerative Diseases and UCSF Weill Institute for Neurosciences, University of California San Francisco, San Francisco, CA 94143, USA

[*] These authors contributed equally to the work
✉, Corresponding Authors
LM: lisam@trp.ucsf.edu, DAA: agard@msg.ucsf.edu

Results

Phosphorylation at position 129 is the major ASyn modification accumulated in Lewy bodies from Lewy Body Disease patients(2) and this modification is used to track ASyn aggregate formation in cell and animal models. We found that the previously reported ASyn oligomerization cell model using overexpression of split gLuc tags on ASyn(4) drives ASyn into large S129 phospho-ASyn-containing aggregates in H4 neuroglioma cells, whereas untagged ASyn or ASyn tagged with Turquoise fluorescent protein does not (Fig S1). As the presence of such aggregates could complicate investigation of oligomers in cells, we tested the split NanoBit(5)(nLuc) tags on ASyn that we designed for the biochemical assay described in the main text for the formation of PO_4-ASyn aggregates. ASyn fused at its C-terminus with either the large (LgBit) or the small (smBit) portion of nLuc or a mixture of the two formed few, if any ASyn PO_4-positive aggregates (Fig S1). This enabled us to establish a quantitative cellular ASyn oligomerization assay using the split nLuc-tagged ASyn system. Split nLuc tags placed on separate ASyn molecules reconstituted luciferase activity, whereas removal of ASyn from one of the tags gave minimal background signal (Fig S2). We established conditions in which nLuc activity resulting from complementation of split nLuc tags attached to ASyn is proportional to the amount of tagged ASyn transiently overexpressed in H4 neuroglioma cells in both cellular and media compartments (Fig S2). Establishing a proportional assay required optimal nLuc substrate type and concentration, optimal transfection conditions, use of optimum medium, and optimal

delay and integration times in reading the luciferase activities. In order to use Hsp70 variants to probe mechanisms, we needed to show they did not artificially perturb cells. We show here that overexpression of the Hsp70 variants and the split nLuc tagged ASyn did not impact cell densities (Fig S3).

Figures and Legends

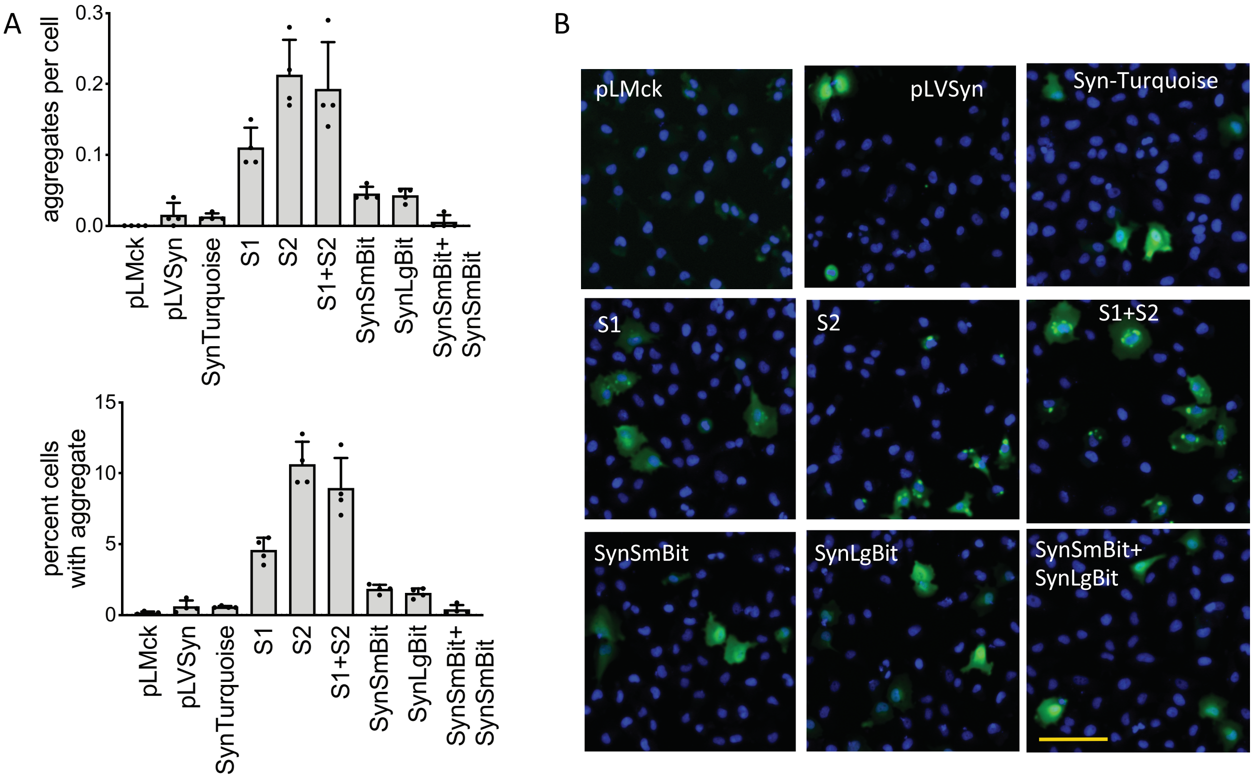

Figure S1. Split Gaussia but not split nLuc tags on ASyn drive S129 PO_4 positive aggregates. H4 cells were transfected with the indicated plasmids, fixed and stained, and aggregates containing S129-phosphorylated ASyn (PO_4-ASyn) quantitated on an Arrayscan automated imaging system as described in methods. A) Quantitation of aggregates per cell (top) and the percentage of cells with aggregates (bottom) indicates that expressing split Gaussia luciferase-tagged ASyn (S1 and S2) generates abundant aggregates, whereas vectors expressing nLuc split tags (SynSmBit and SynLgBit) have few to none. Control empty vector (pLMck), untagged ASyn (pLVSyn) and ASyn tagged with the fluorescent protein Turquoise (plasmid pCKSynT1, SynTurquoise) have no aggregates. Means ±s.d., n=4 wells. B) Representative images from each plasmid showing nuclei (blue) and PO_4-ASyn (green). Scale bar = 100 μM.

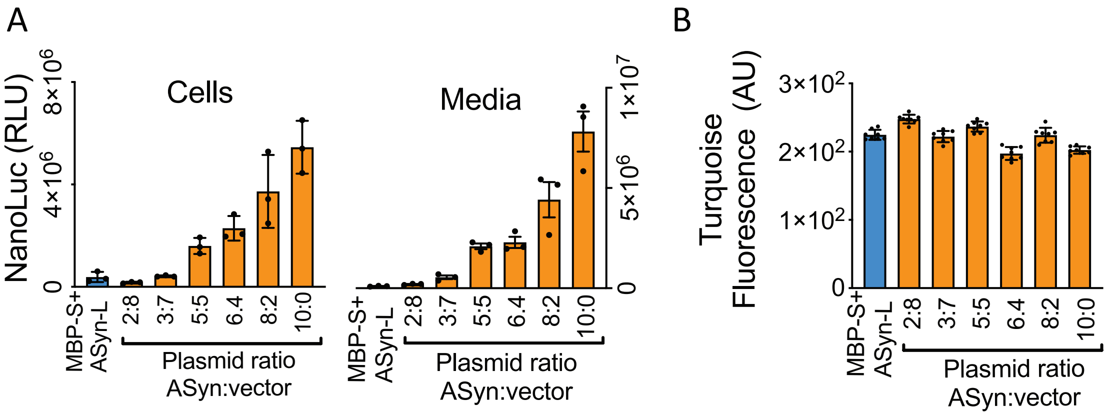

Figure S2. Development of a cellular ASyn oligomerization assay.
Control H4 cells were transfected with equal amounts of maltose-binding protein fused with nLuc small bit (MBP-S, plasmid pckMBP-LgBit) and ASyn fused to nLuc large Bit (ASyn-L, plasmid pckSyn-LgBit) (blue). An equal mixture of plasmids pckSyn-SmBit (ASyn-S) and pckSyn-LgBit (ASyn-L) were diluted with non-expressing vector (orange) as indicated and transiently transfected into H4 cells as described in methods. The 10:0 ratio is equivalent to the amounts of split nLuc tags in the control (blue) samples. A trace (1/10) amount of control vector expressing Turquoise fluorescent protein (pCKSynT1) was included for each sample to measure overall transfection efficiency. 48 hours post transfection cells were assayed for Turquoise fluorescence and cells and media were assayed for nLuc activity as described in methods. nLuc activity is dependent on presence of a mixture of both tagged forms of ASyn. The amount of nLuc measured is proportional to the amount of ASyn plasmid transfected for both cells and media. Turquoise controls indicate reproducible transfection efficiencies between samples. Means ± s.d. n= 3.

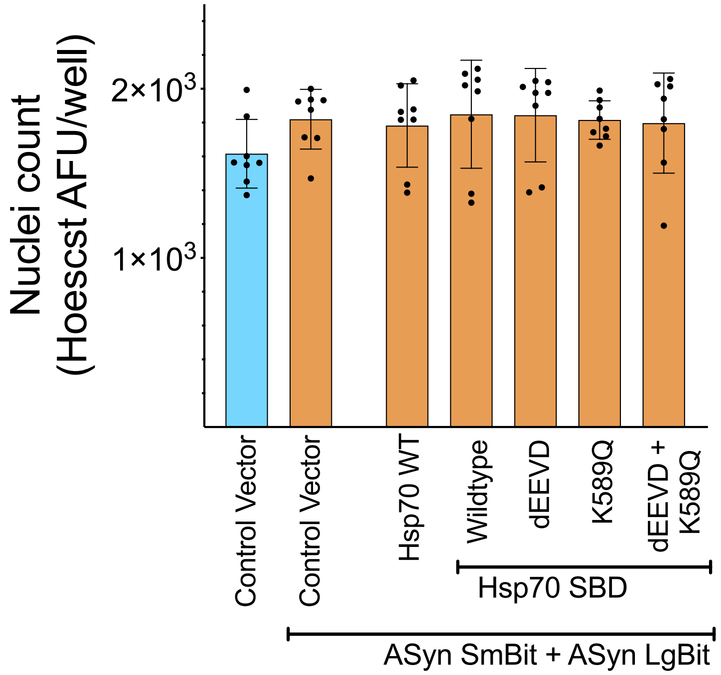

S3. Lack of toxicity of transfected ASyn and Hsp70 constructs.
H4 cells were transfected with control non-expressing pVLBobi vector (blue), with a mixture of plasmids pckSyn-SmBit (ASyn-SmBit) and pckSyn-LgBit (ASyn-LgBit) (orange) and with the indicated variant of Hsp70 (Hsp70 WT is full length wildtype) as described for JetPrime transfection in the methods of the main paper. Two days later the nuclei are stained with Hoechst dye and Hoechst fluorescence quantitated as a surrogate for cell number. Overexpression of none of the Hsp70 variants showed impact on cell densities. Means ± s.d. n= 3.

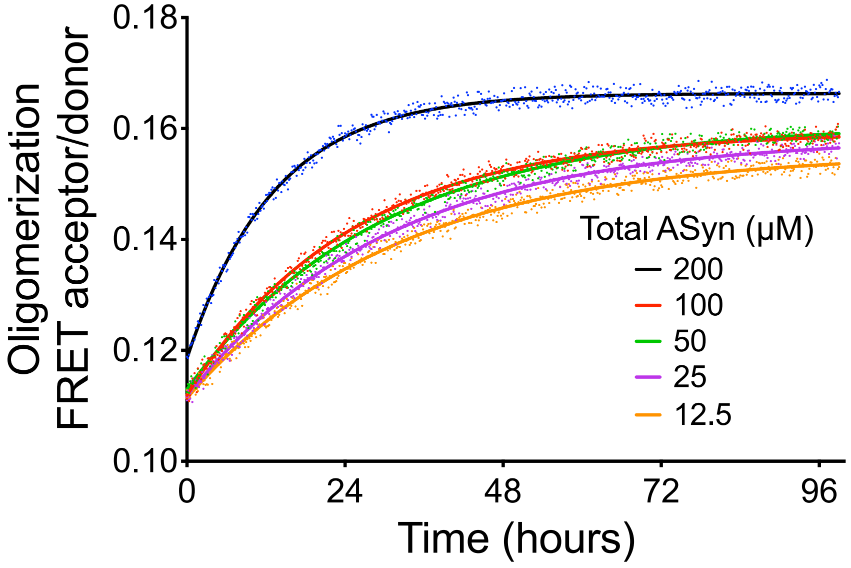

S4. The dependence of the FRET oligomerization assay on ASyn concentrations.
A FRET-based ASyn oligomerization assay was performed at various concentrations of ASyn as reported(1). The means of three replicates and first-order kinetic fit (line) calculated using Prism software are shown. The initial rates calculated from these data were used to generate Fig 1F in the main manuscript. A subset of these data was shown in a prior publication using a different graphing format(1) and is licensed under the Creative Commons Attribution 4.0 International License at https://creativecommons.org/licenses/by/4.0/(3).

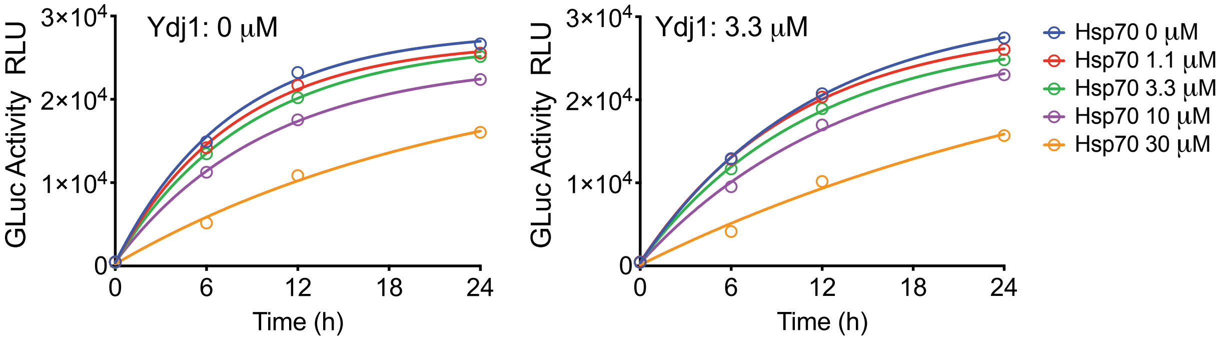

S5. Hsp70 impairment of ASyn oligomerization is not influenced by J protein.

The split Gaussia luciferase (GLuc) based oligomerization assay was performed as described in methods of the main manuscript using 1 mM ATP. Hsp70 at various concentrations were tested in the presence or absence of 3.3 μM Yeast J protein YDJ1. J proteins enhance ATP hydrolysis. Consistent with the lack of dependence on ATP cycling, there is no impact of YDJ1 on Hsp70 inhibition of ASyn oligomerization.

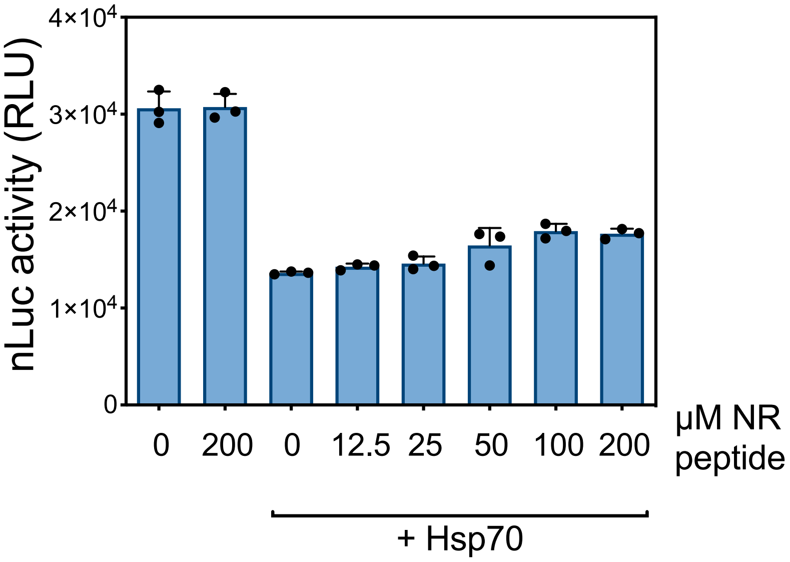

S6. NR peptide does not impair Hsp70 blockage of ASyn oligomerization at multiple concentrations.

10 μM each of ASyn-LgBiT and ASyn-SmBiT in PBS containing 1 mM MgADP, were incubated with or without 20 μM full-length Hsp70, and various concentrations of the NR peptide for 24 hours and nLuc luciferase activity measured. NR peptide does not block Hsp70 action on ASyn oligomerization at concentrations at or above its Kd (Figure 4D of main paper).

Methods

Molecular cloning

PLVSyn, a gift from Brian Spencer, expresses untagged wild-type human ASyn driven from the CMV promoter in the lentivirus vector described(6). S1 and S2, gifts from Pamela McLean, express ASyn fused to the N and C terminal split fragments of Gaussia luciferase respectively(4). pLMck1 expression vector was generated by inserting into plasmid pcdna3.1+ the hybrid adenovirus MLP IgG splice sequence shown to enhance expression in mammalian cells(7) at the 5 prime end of the multi cloning site. The following vectors express the indicated proteins in the vector pLck1: pckSyn-SmBit and pckSyn-LgBit express ASyn fused at its C-terminus with a linker followed by the small bit or large bit respectively of split nLuc as described for our bacterial expression vectors. pckMBP-LgBit expresses the maltose-binding protein fused at its C-terminus with the large bit of the nLuc system(5) with the linker sequence IDGGGGSGGGGSSG between the two proteins. PCK_T1 expresses fluorescent protein mTurquoise2(8). pCKSynT1 expresses ASyn tagged at its C-terminus with the linker GSAAPVATGS followed by mTurquoise2 coding sequence. Additional plasmids are described in the methods of the main paper.

Biochemical ASyn oligomerization assays

The split Gaussia luciferase (gLuc) based oligomerization assay was performed as described(1), with the additions for Hsp70 impact noted below. 10 µM each of αSyn-GLuc1 and αSyn-GLuc2 (N-terminal or C-terminal fragment of GLuc fused to the C-terminus of αSyn) were incubated in a buffer containing 1 mM $MgCl_2$, 1 mM ADP (Fig 3A) or ATP (fig S5), 50 mM Na_3PO_4, 0.02% NaN_3 and 1 mM DTT at pH 7.4 at 37°C in a thermal cycler (BioRad DNA Engine PTC-200). Hsp70 or Hsp70 plus YDJ1yeast J protein at various concentrations were added at the beginning of the assay. At 24 hours of incubation GLuc activity was quantified using the BioLux Gaussia Luciferase Assay Kit (NEB) according to the manufacturer's instructions with luminescent detection on a SpectraMax M5 Microplate Reader (Molecular Devices) using a 5-minute delay after the addition of substrate and 3 seconds of integration time.

The Forster resonance energy transfer (FRET) oligomerization assay was carried out as described(1)10 µM each of ASyn-Q99C-Cy3 (donor) and ASyn-Q-99C-Cy5 (acceptor) in PBS (pH 7.4) containing 1 mM ADPand 1 mM MgCl2 were dispensed into black/clear bottom 384-well plates (Greiner) using a Mantis liquid handler (Formulatrix). The plate was sealed with AbsorbMax film (Sigma-Aldrich) and incubated at 37°C. The FRET between donor and acceptor was measured on a SpectraMax M5 Microplate Reader (Molecular Devices). An excitation wavelength of 525 nm for ASyn-Cy3 (donor), and emission wavelengths of 570 nm and 670 nm for ASyn-Cy3 (donor) and αSyn-Cy5 (acceptor) respectively were used. The plate reader PTM sensitivity was set to medium and assays executed without mixing. The signals were read at 10-minute intervals for 99 hours at 37°C. Efficiency of FRET was calculated as follows: $E_{FRET}=I_{Acceptor}/I_{Donor}$, in which $I_{Acceptor}$ is acceptor emission intensity, I_{Donor} is donor emission intensity. Hsp70 at various concentrations were added in Fig 3C.

Cellular nLuc assay
H4 cells are plated at $7x10^3$ cells per well in a polyD-Lysine-coated clear-bottom white 96-well plate in DMEM plus 10% FCS and the next day were transfected with DNA and Fugene (Promega, Madison, WI) as per manufacturer's recommendations using a ratio of transfection reagent to DNA of 4:1. 24 hours after transfection the media was replaced with Opti-MEM without phenol red medium (Opti-MEM). The next day, as a surrogate measure of cell densities, the plates were read on a microplate reader SpectraMax M5 (Molecular Devices LLC,) with an excitation wavelength of 420 nm and emission wavelength of 480 nm to determine Turquoise fluorescence levels. To harvest the media, the plates were spun for 5 minutes at 1050 rpm in an Eppendorf, model 5804 and 40 μl of supernatant was transferred to a 96-well white plate and mixed with 40 μl Opti-MEM. Cells were washed in PBS and lysed in 30 μl of cell lysis buffer (luciferase cell lysis buffer, NEB Catalog # B3321) at room temperature and mixed on an orbital shaker for 30 minutes. Oligomerization was quantified by measuring the luminescence on a SpectraMax L Microplate Reader (Molecular Devices). 80 µl for media or 100 µl for cells of Coelenterazine (CTZ) native substrate (40 µM, NanoLight Technology) in Opti-MEM was injected into the wells with the samples using the injector integrated in SpectraMax L. nLuc activity was measured using a 0.1-second delay after the addition of substrate and a 2-second integration time. Additional cell assays are described in the methods of the main paper.

Cell staining and Imaging
H4 cells were plated at $7x10^3$ cells per well in DMEM with 10% FCS in a polyD-Lysine-coated μclear black 96-well plate (Greiner Bio-One, Monroe, NC) and the next day were transfected with DNA and Fugene (Promega, Madison, WI) as per manufacturer's recommendations using a ratio of transfection reagent to DNA of 4:1. The following day, the media was replaced with fresh media. 72 hours after transfection cells were fixed for 15 minutes in 4% PFA. Cells were stained with 1 µg/ml of purified S129-phosphorylated ASyn specific antibody 11A5(2) overnight at 4^oC. They were washed 3 times in PBS and incubated with a goat anti-mouse secondary antibody (Invitrogen) at 1/300 concentration and Hoescht 33342 (Invitrogen) diluted at 1/2000 to stain nuclei for 2 hours at room temperature. Cells were then washed 3 times in PBS. Nuclei and ASyn aggregates were imaged on an Arrayscan XTI high content automated imaging system (Thermo Fisher, Cellomics) and analyzed for aggregates using the spot-detector paradigm of the HCS Studio™ Cell Analysis Software.

References

1. Toth, G., Neumann, T., Berthet, A., Masliah, E., Spencer, B., Tao, J., Jobling, M. F., Gardai, S. J., Bertoncini, C. W., Cremades, N., Bova, M., Ballaron, S., Chen, X. H., Mao, W., Nguyen, P., Tabios, M. C., Tambe, M. A., Rochet, J. C., Junker, H. D., Schwizer, D., Sekul, R., Ott, I., Anderson, J. P., Szoke, B., Hoffman, W., Christodoulou, J., Yednock, T., Agard, D. A., Schenk, D., and McConlogue, L. (2019) Novel Small Molecules Targeting the Intrinsically Disordered Structural

Ensemble of alpha-Synuclein Protect Against Diverse alpha-Synuclein Mediated Dysfunctions. *Sci Rep* **9**, 16947

2. Anderson, J. P., Walker, D. E., Goldstein, J. M., de Laat, R., Banducci, K., Caccavello, R. J., Barbour, R., Huang, J., Kling, K., Lee, M., Diep, L., Keim, P. S., Shen, X., Chataway, T., Schlossmacher, M. G., Seubert, P., Schenk, D., Sinha, S., Gai, W. P., and Chilcote, T. J. (2006) Phosphorylation of Ser-129 is the dominant pathological modification of alpha-synuclein in familial and sporadic Lewy body disease. *J Biol Chem* **281**, 29739-29752
3. Creative Commons license. https://creativecommons.org/licenses/by/4.0/
4. Danzer, K. M., Ruf, W. P., Putcha, P., Joyner, D., Hashimoto, T., Glabe, C., Hyman, B. T., and McLean, P. J. (2011) Heat-shock protein 70 modulates toxic extracellular alpha-synuclein oligomers and rescues trans-synaptic toxicity. *Faseb J* **25**, 326-336
5. Dixon, A. S., Schwinn, M. K., Hall, M. P., Zimmerman, K., Otto, P., Lubben, T. H., Butler, B. L., Binkowski, B. F., Machleidt, T., Kirkland, T. A., Wood, M. G., Eggers, C. T., Encell, L. P., and Wood, K. V. (2016) NanoLuc Complementation Reporter Optimized for Accurate Measurement of Protein Interactions in Cells. *ACS Chem Biol* **11**, 400-408
6. Marr, R. A., Rockenstein, E., Mukherjee, A., Kindy, M. S., Hersh, L. B., Gage, F. H., Verma, I. M., and Masliah, E. (2003) Neprilysin gene transfer reduces human amyloid pathology in transgenic mice. *J Neurosci* **23**, 1992-1996
7. Huang, M. T., and Gorman, C. M. (1990) Intervening sequences increase efficiency of RNA 3' processing and accumulation of cytoplasmic RNA. *Nucleic Acids Res* **18**, 937-947
8. Goedhart, J., von Stetten, D., Noirclerc-Savoye, M., Lelimousin, M., Joosen, L., Hink, M. A., van Weeren, L., Gadella, T. W., Jr., and Royant, A. (2012) Structure-guided evolution of cyan fluorescent proteins towards a quantum yield of 93%. *Nat Commun* **3**, 751
